# Patterns and associates of cognitive function, psychosocial wellbeing and health in the Lothian Birth Cohort 1936

**DOI:** 10.1186/1471-2318-14-53

**Published:** 2014-04-23

**Authors:** Andrea R Zammit, John M Starr, Wendy Johnson, Ian J Deary

**Affiliations:** 1 Centre for Cognitive Ageing and Cognitive Epidemiology, Edinburgh, UK; 2 Albert Einstein College Medicine, New York, USA; 3 Centre for Cognitive Aging and Cognitive Epidemiology, Department of Psychology, University of Edinburgh, 7 George Square, Edinburgh EH8 9JZ, Scotland, UK

## Abstract

**Background:**

Cognitive function, psychosocial wellbeing and health are important domains of function. Consistencies and inconsistencies in patterns of wellbeing across these domains may be informative about wellbeing in old age and the ways it is manifested amongst individuals. In this study we investigated whether there were groups of individuals with different profiles of scores across these domains. We also aimed to identify characteristics of any evident groups by comparing them on variables that were not used in identifying the groups.

**Methods:**

The sample was the Lothian Birth Cohort 1936, which included 1091 participants born in 1936. They are a community-dwelling, narrow-age-range sample of 70-year-olds. Most had taken part in the Scottish Mental Survey 1947 at an average age of 11, making available a measure of childhood intelligence. We used latent class analysis (LCA) to explore possible profiles using 9 variables indicating cognitive functioning, psychosocial wellbeing and health status. Demographic, personality, and lifestyle variables – none of which were used in the LCA – were used to characterize the resulting profile groups.

**Results:**

We accepted a 3-group solution, which we labeled High Wellbeing (65.3%), Low Cognition (20.3%), and Low Bio-Psychosocial (14.5%). Notably, the High Wellbeing group had significantly higher childhood IQ, lower Neuroticism scores, and a lower percentage of current smokers than the other 2 groups.

**Conclusion:**

The majority of individuals were functioning generally well; however, there was evidence of the presence of groups with different profiles, which may be explained in part in terms of cognitive ability differences. Results suggested that higher life-long intelligence, personality traits associated with less mental distress, and basic health practices such as avoiding smoking are important associates of wellbeing in old age.

## Background

The World Health Organization defines health in terms of wellbeing across physical, cognitive and social domains of function [1]. Wellbeing in old age is also typically defined as high function across these three domains [2-7]. In a recent review [4] of the measures used to operationalize successful aging, the authors found that the most commonly used variables to define wellbeing in old age were physical fitness, cognitive functioning, social wellbeing, and life satisfaction, in that order. They also found that a mixture of standardized tests (such as grip-strength or the Mini-Mental State Exam, MMSE) and self-report measures (such as activities of daily living, ADLs) was present in most studies. Furthermore, cross-sectional work on wellbeing in older adults increasingly shows that, although some individuals show good or poor wellbeing consistently across all studied measures, others function relatively well in some measures but poorly in others [8-14].

Recent studies have sought to characterize profiles across specific areas of wellbeing in old age as a first step in understanding individual differences in aging e.g. [9,11,13-16]. For example, Pruchno et al. [16] investigated physical function in individuals between the ages of 50 and 74 using both objective (physical function and disease) and subjective (self-rated health) indicators. They found that one group performed well on both objective and subjective indicators, another on subjective indicators only, another on objective indicators only, and the last group did not do well on either objective or subjective indicators. Using data from the Berlin Ageing Study (BASE), Smith & Baltes [12] also used both objective and self-rated measures to investigate cognitive ability, personality, and social wellbeing in individuals between the ages of 70 and 103 [17]. They identified 9 different profiles of psychological functioning. Most individuals had at least moderately positive profiles, including good cognitive ability, high extraversion, and high engagement in family and social projects. However, many individuals showed profiles that were not consistently ‘good’ or ‘bad’ across variables. For example, one group had high cognitive function and loneliness, but low extraversion and little belief that their actions could influence what happened to them. Another group’s (rather contradictory) characteristics included high neuroticism, high-perceived control and high belief that actions of other people would determine what happened to them.

Hsu and Jones [5] studied chronic disease and physical function, emotional support, depressive symptoms, social participation, and social satisfaction in individuals over the age of 50. They identified four groups: a successful group (i.e. free from disease and with high cognitive and physical function), a usual group (i.e. with age-related limitations), a group with poor health and another that is dependent on care support. Functional disability in old age has also been investigated using instrumental activities of daily living (IADLs), physical activities of daily living (PADL), physical activity, and sensory, mobility and cognitive domains of function [18]. The authors found three groups: one was high functioning, another had IADL deficits only, and the last group displayed multiple disabilities. Fiori and Jager [9], Fiori et al. [10]; Fiori et al. [19] and Litwin [20] used social network measures from various datasets including the Wisconsin Longitudinal Study, Americans’ Changing Lives Study, the Berlin Aging Study, and data from the Israeli Central Bureau of Statistics to classify types of individuals’ social networks in old age. Their results varied from between five and six types of networks with differing definitions of networks (such as family- or friends-focused), levels of support (e.g. restricted, unrestricted) and satisfaction. Using the same sample as in the present study, our group examined patterns of three self-rated aspects of psychosocial function in the Lothian Birth Cohort 1936 [14]. We identified 5 groups; of these, 3 groups performed either better, about the same as, or poorly relative to mean levels across all three self-reported measures. The other 2 groups displayed uneven profiles of psychosocial wellbeing, with much better function in one area than in another. One of these groups reported high physical activity but low emotional wellbeing; the other group reported the opposite pattern. From this point on we use the term ‘uneven profile’ to refer to this kind of disparity in level of function across functional domains.

These studies have also identified characteristics that are associated with certain groups or patterns. That is, they used some variables to identify groups or characterize profiles, and then used new sets of variables to describe them further. Generally, participants with more desirable (i.e. displaying high scores on areas of function) profiles also had more years of education, better health, and better physical wellbeing [12], emphasizing consistency in areas of wellbeing; however, some participants fell into groups with uneven patterns of wellbeing, providing information on the characteristics of certain subtypes which may need more attention.

This literature demonstrates that wellbeing in old age across domains of function can take various forms, and it typically highlights three main areas of function important for high overall wellbeing – high cognitive function, high physical function and physical health, and good psychosocial wellbeing [4,6,21,22]. Most studies, however, have focused on only one domain of function. Lovden et al. [23], only studied cognitive function. Some studied two domains. For example, Andrews et al. [2], studied physical and social function; and Morack et al. [24] studied psychosocial and cognitive function. A few adopted broader approaches using multiple domains. For example, Smith & Baltes [12] studied cognitive function, personality, health, and social inclusion; Ko et al. [11] studied cognition, health, personality and social support. Some used only one data collection format such as self-report [7,14,25,26] while others used both subjective and objective measures [16,27]. There were differences in results, perhaps because of such differences in breadth and type of data. Furthermore, so far no studies have simultaneously addressed domains of cognitive function, psychosocial wellbeing and health fitness that included both objective and self-report measures across a study population. Thus, application of multiple markers of wellbeing in models of potentially differential wellbeing has not been thoroughly implemented. Attempting to identify groups of individuals using models of wellbeing has resulted in mixed views on the identification (if any) of patterns of wellbeing in old age.

Most studies have used variable-oriented approaches: they have examined how wellbeing-associated variables were correlated and to what other variables they related. Studies with person-oriented approaches to wellbeing in old age are still in their early stages [28,29]. Variable-oriented studies have shown that some domains of function are highly correlated such as physical and cognitive function, psychosocial and emotional wellbeing, and even cognitive and emotional wellbeing [30-41]. Variable–oriented studies have also shown that high cognitive function is an important associate of autonomy, physical health, functional independence, quality of life, and low levels of disease and mortality [31,36,41,42] and that poor physical health, functional limitations, physical disability or dependence on others are associated with feelings of depression [43-45]. These studies are valuable in providing the correlates of wellbeing; however, they do not provide information on how individuals are grouped across these variables.

In person-oriented or group-oriented studies, where participants are the main focus of the study; variables are used to discover whether there are discernable groups of people who differ in patterns of wellbeing. Typically, person-oriented studies employ cluster or latent class analysis to extract groups of individuals based on a number of core variables of interest. For example, Smith and Baltes [29] used cluster analysis to extract groups of individuals based on scores on social, cognitive, personality and health measures in the Berlin Aging Study. Ko et al. [11] used latent profile analysis to extract groups on measures of cognitive function, social support and personality in the Health and Aging Study. Both of these methods produce a number of subgroups defined by the researcher; then the analysis assigns cases to the group they have the highest probability of belonging. Different studies generate different number of groups, mainly because there are no specific grouping criteria, and because different samples have different variables. In person/group-oriented analyses variables that are not used in the core analysis to extract the groups such as demographic and lifestyle variables can provide further useful information about the group characteristics. For example Smith and Baltes [29], used these ‘external’ variables—those not used in the cluster analysis and external to the core variables they were using—, such as education, physical functionality, and medical illness, to characterize further the profiles they extracted. They found that the higher-functioning profiles also had more years of education, and were in better health and physical condition. In our previous study we also used a person/group-oriented approach, but we explored only one domain of function commonly depicted in the literature on wellbeing in old age – psychosocial wellness [14]. Although this is an important and valued component recognized by the literature and by older individuals themselves [4,22] it excludes vital information on the status of cognitive and physical function in old age.

In this study we added to our previous work on emotional and psychosocial wellbeing by exploiting current definitions of the constituents of wellbeing that are widely depicted in literature on old age [4,6,21,22,25,46]. We extended our previous model [14] substantially by adding two more domains to our exploration of patterns/profiles of function in old age: namely, cognitive function and health. Furthermore, the variables that we used in the previous study were all self-rated measures relating to psychosocial and emotional wellbeing. Here we included objective cognitive ability tests and health measures, as well as subjective measures relating to psychosocial and emotional wellbeing. By combining health, cognitive, and psychosocial components, our intention was to analyze wellbeing in old age according to broader definitions of domains involved in successful aging [4,6,21,22,25,47,48] that included structured assessments of cognitive function and health as well as self-rated measures on psychosocial and emotional variables. In this cross-sectional study we extended our profile analysis from our previous work to a broader definition of ageing that included cognitive function and health. Our aims were: i) to investigate whether there are groups of individuals with different profiles of scores across domains of cognitive function, psychosocial wellbeing and health; ii) to identify characteristics of any evident groups by comparing them on demographic, personality, and lifestyle variables that were not used in identifying the groups.

**Figure 1:**
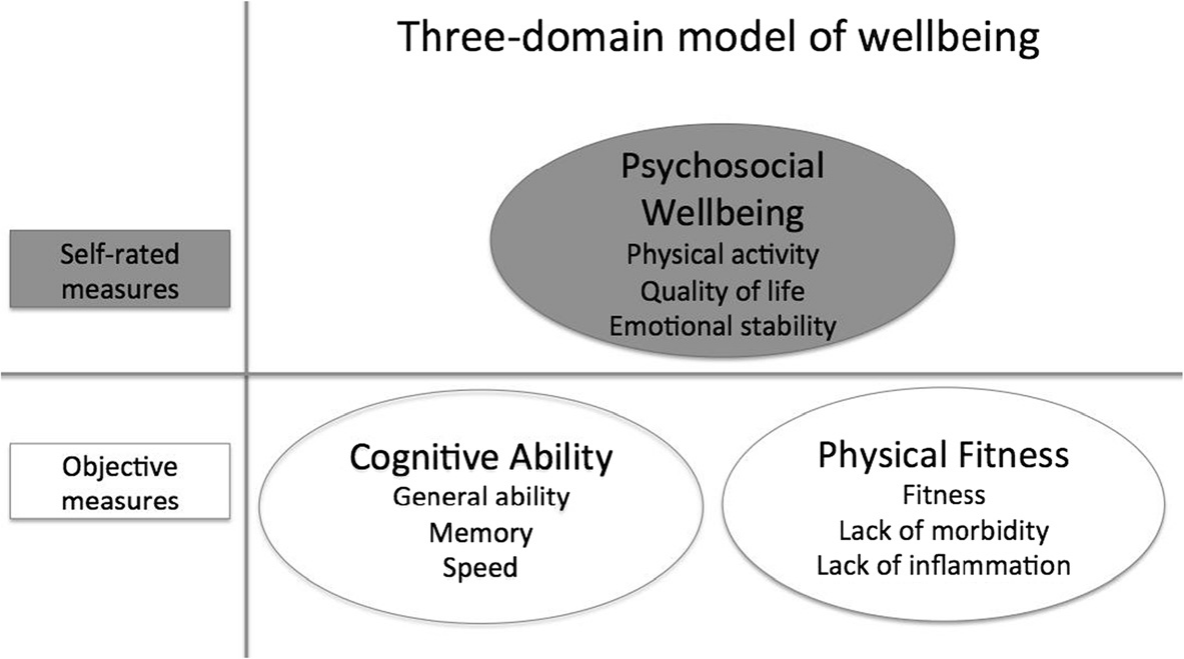
**The model we used to study wellbeing in the Lothian Birth Cohort 1936, including three components and each of the variables making up these components to measure each of the three domains of wellbeing.** The variables in the shaded domains illustrate self-rated measures, which were used in Zammit et al. [14], whilst the variables in the white domains illustrate objective-based measures, which we added in this study.

Figure 1 illustrates our model. In our study we first applied LCA to the three domains of function to explore whether different profiles/patterns of wellbeing were discernable. Secondly, we explored external associates of any such profiles. That is, we examined the groups’ associations with a set of variables, not used in the formation of the profiles. These included demographic factors, prior cognitive ability, personality traits, and health behaviors. The availability of early childhood IQ in this study was particularly valuable because some of the measures used in this study, such as cognitive ability and better health in old age have been found to be associated with childhood IQ [49-52]. We tested whether early childhood IQ was predictive of various aspects of later-life wellbeing. It is reasonable to think that IQ might be associated with wellbeing in old age because of its capacity to predict morbidity and mortality [49,50] both of which are associated with other characteristics, including educational and occupational outcomes, health and lifestyle choices also associated with IQ, as well as with personality. Individuals with higher IQ may have sets of skills that may help in delaying disease and avoiding death [50].

On the basis of our previous study [14] we were aware that the majority of the sample would demonstrate high scores across all three domains of function; we hypothesized, however, that smaller subgroups with uneven patterns across domains would emerge. The smaller subgroups were hypothesized to have distinctive profile characteristics in external variables measuring demographic, personality and lifestyle variables.

## Methods

### Sample

The study’s sample consisted of members of the Lothian Birth Cohort 1936 (LBC1936), which included 1091 participants (548 males and 543 females) born in 1936 and mostly living in the Edinburgh area of Scotland at about age 70 years. Most had taken part in the Scottish Mental Survey 1947 at an average age of 11, making available a measure of childhood cognitive ability. They were assessed between 2004 and 2007, when they had a mean age of 70 years [51,53]. The study was in compliance with the Helsinki Declaration, and ethical permission was obtained from the Multi-Centre Research Ethics Committee for Scotland (MREC/01/0/56) and from the Lothian Research Ethics Committee (LREC/2003/2/29). All participants gave informed consent in writing. Detailed methodological description is available in Deary et al. [51] for all the measures listed below.

We studied the domains of physical health status, cognitive function, and psychosocial wellbeing. Three components were formed within each of these domains, and each of these components was formed from multiple variables using principal components analysis. A list and description of the variables we used to make up each of these components for each domain is provided next, followed by the external covariates we used to characterize any resulting groups.

### Measures of the Domains of Wellbeing

#### Cognitive ability domain components

Here, we assessed three aspects of cognitive function that have emerged empirically [40] as being important in older age.

##### I. General cognitive ability (g)

We used six Wechsler Adult Intelligent Scale-III^UK^ (WASI-III [54]) subtest scores to derive *g* or general intelligence; these included Symbol Search, Digit-Symbol Coding, Matrix Reasoning, Digit-Span Backwards, Letter-Number Sequencing, and Block-Design.

##### II. Memory

We used four subtests from the Wechsler Memory Scale-III^UK^ (WMS-III [55]), which included Logical Memory I (immediate recall of verbal declarative memory); Logical Memory II (delayed recall of verbal declarative memory); Verbal Paired Associates I (immediate verbal learning memory); and Verbal Paired Associates II (delayed verbal learning memory) to derive the memory component.

##### III. Processing Speed

We used means of Simple Reaction Time (SRT) and Choice Reaction Time (CRT) [56,57] and Inspection Time (IT; non-speeded elementary visual processing assessed on a computer [58]; to derive this component.

#### Psychosocial wellbeing domain components

This is the domain that we investigated on its own in our previous study of this cohort [14]

##### I. Physical functioning

We used three variables: self-reported level of physical activity, total number of days active per month, and activities of daily living to derive this component. Participants reported their level of physical activity on a 6-point scale of intensity varying from house-chores (low intensity) to intense exercise (high intensity), depending on how often they engaged in such activities for more than twenty minutes at a time per month. For the second variable, total number of days active per month, participants were asked how many days they engaged in vigorous exercise that lasted more than twenty minutes at a time per month. Hence the first variable measured level of intensity and the second frequency of activity. Higher figures indicated higher levels of activity in both instances. For activities of daily living, participants completed the Townsend scale (1979) [59], a 9-item scale that assesses ability to perform activities involved in personal hygiene, getting dressed, eating independently, and being mobile. Answers could range from ‘yes, with no difficulty’, to ‘yes, with some difficulty’, and ‘no, needs help’, with scores of 0, 1, and 2 respectively. The scores for the Townsend scale were reversed so higher scores characterized higher ability to perform activities independently.

##### II. Emotional wellbeing

We used the Hospital Anxiety and Depression Scales (HADS) [60] to measure emotional wellbeing. This assesses recently-prevailing emotional states. There are seven items for anxiety and seven items for depression, with scores ranging from 0 to 3 per item, and 0 to 21 per subscale. Because PCA generally requires at least 3 variables, we standardized the two sub-scores and calculated their mean. Higher scores signify greater anxiety or depression. These scores were reversed so that higher scores characterized more positive emotional wellbeing.

##### III. Quality of Life (WHOQOL-BREF) Assessment

Participants completed the brief version of the World Health Organization Quality of Life (WHOQOL-BREF) Assessment [61]. This measures self-reported quality of life. There are 26 questions in all covering: mental health; social, emotional and physical role functioning; general health perceptions; bodily pain; physical function; and vitality. All items are measured on a five-point scale, with higher scores denoting better quality of life. This questionnaire has good validity, reliability and consistency, and is applicable cross-culturally [61].

#### Health Status domain components

We chose three quite distinct domains of health: three measures of fitness, three blood-based measures of inflammation, and history of illness and medication.

##### I. Fitness

We measured grip strength in both left and right hands using a North Coast Hydraulic Hand Dynamometer (JAMAR). The best of three attempts with each hand was recorded and then averaged. Forced expiratory volume from the lungs in 1 second was calculated using the best of three trials using a Micro Medical Spirometer. 6-meter walk-time required participants to walk 6 meters as quickly as possible to do safely. The time taken to walk the distance was the score. All three variables were adjusted for weight and height. We used the inverse of the 6-metre walk-time to equate higher scores with faster gait speed.

##### II. Inflammation

Inflammation markers have shown associations between their presence in plasma levels and development of dementia [62,63], depression [64,65] and cardio-vascular disease [66]. We measured inflammation using C-Reactive Protein (CPR), Neutrophil Count, and Fibrinogen from blood samples. CRP was tabulated using a dry-slide immuno-rate method on OrthoFusion 5.1 F.S. analyzers. Fibrinogen was measured using an automated Clauss assay (TOPS coagulometer, Instumentation Laboratory, Warrington, UK). Neutrophil count was completed using the absolute neutrophil count (ANC) calculator. We reversed the scores on this composite so that a higher score would indicate better wellbeing; we renamed the variable Lack of Inflammation.

##### III. Morbidity

Participants were asked if they had histories of high blood pressure, diabetes, high cholesterol, cardiovascular disease, leg pain, blood circulation problems, stroke, cancer, thyroid, Parkinson’s disease, arthritis, gout, or any other diseases. They also reported all medications they were taking at the time. We standardized the variables, and their mean was calculated; we then reversed the counts so that a higher composite score would indicate better wellbeing, renaming the variable Lack of Morbidity.

#### External variables not used to identify the groups

##### Demographic measures

These included self-reported total number of years in formal education, marital status (i.e., single, married, widowed, separated, or divorced), whether or not living alone, and highest professional social class during working life. The latter was based on Her Majesty’s Stationary Office rankings, ranging from I, which is the most professional social class, to V, which is the most manual class. Class III is divided into non-manual and manual groups [67]. For females, husband’s social class was used when this was higher (more professional). Scores were revered for the correlation matrix so that higher scores indicated higher social class.

##### Prior cognitive ability

The Moray House Test No. 12 [68,69] is a test of general cognitive ability that was administered when participants were aged about 11 years, on 4th June 1947, as part of the Scottish Mental Survey 1947. It includes same-opposites, word classifications, analogies, and practical, reasoning, proverbs, arithmetic, spatial, mixed sentences, and cipher-decoding items. The National Adult Reading Test (NART) [70] is widely used to estimate prior cognitive ability. It requires the participant to read 50 irregular English words aloud. The latter was administered as part of the age-70 LBC1936 assessment.

##### Personality measures

The NEO Five Factor Inventory (NEO-FFI [71]) was used to measure personality traits. This is a 60-item inventory assessing five major personality factors: Neuroticism, Extraversion, Openness to Experience, Agreeableness, and Conscientiousness. Neuroticism reflects lack of emotional stability, such as tendencies toward anxiety and depression. Extraversion reflects sociability, such as time spent with others and enjoyment of talking to people. Openness to Experience reflects the disposition to entertain novel ideas and engage in intellectual pursuits. Agreeableness reflects sensitivity to smooth relations with others and empathy. Conscientiousness reflects self-discipline and self-control. Participants rated the applicability of each item to themselves on a five-point Likert scale, ranging from strongly disagree to strongly agree.

##### Health behavior

Participants reported current alcohol consumption per week and whether they were current or former smokers, or had never smoked. Although body mass index (BMI; kg/m^2^) is not a health behavior per se, it was included in this section since it reflects some direct health practices, such as dietary and exercise habits.

#### Procedure

First, we reduced the variables to be used in the LCA that were relatively homogeneous into components to represent each domain. We derived these components using principal components analysis (PCA), except in the few exceptions mentioned above where we used the means of standardized scales. The components included: *g*, memory, and processing speed to represent cognitive ability; quality of life, emotional wellbeing, and physical function to represent psychosocial wellbeing; and physical fitness, lack of inflammation, and lack of morbidity to represent physical health status. We limited outliers to 3 standard deviations from means by trimming them to that level. In each instance, results from the scree plots and eigenvalues suggested one component (total variance for the components ranged from 43.6% to 60%), and the regression scores on the first unrotated principal components were used in the LCA. The use of PCA provided reliable composite variables, and reduced computational demands on the LCA while taking into account several specific aspects of each domain.

Second, we explored the presence of subgroups based on the three domains using latent class analysis implemented in MPlus, version 5.2 [72]. LCA uses general mixture modeling (GMM) techniques to produce a number of subgroups as specified by the researcher [73]. We included all participants despite small amounts of missing data by using the maximum likelihood estimation feature. The variables considered were our principal component scores on *g,* memory, speed, QOL, emotional wellbeing, physical activity, fitness, lack of inflammation, and lack of morbidity dimensions. We specified two-, three-, four-, five-, six-, and seven-group solutions. Participants were then assigned to the group to which they had the highest probability of belonging. In order to identify the solution that seemed to specify the most appropriate number of subgroups we used model-fit statistics. These included the Bayesian information criterion (BIC [74]) and entropy (ENT [75]), which summarized the discrepancy between observed and expected values. Specifically, ENT indicated how well the variables predicted group membership, with ENTs closer to 1 indicating that participants had high probabilities of membership in single groups relative to others, and smaller BICs indicated greater probabilities that the model represented the data, giving substantial weight to model parsimony. Although other model-fit statistics are available, for our sample size and the continuous variables we used, BIC appears to perform the best in identifying the true underlying model and other robust options require substantially greater computation times [76]. Twenty random starts were used in the initial stage and 10 optimisations in the final stage to obtain appropriate model convergence and to be confident of robust solutions. Groups with less than 5% of the sample were avoided because groups of this size are more likely to have resulted from chance sample characteristics [77].

Third, we ran analyses of variance (ANOVAs) with group membership as the independent variable to describe how the groups differed from each other on the demographic, personality and health behavior external variables (i.e. those not used in the LCA-based group formation). Where differences were significant, we applied post-hoc tests using Tukey’s Honestly Significant difference test to identify which groups differed significantly. We used the SDs of the largest group as the bases for calculating effect sizes as the standardized mean differences between the two variables. We did not adjust statistical significance levels for multiple testing in any of these analyses.

Fourth, to describe the differences among the groups more clearly, we applied another PCA to the components we had formed to represent the domains: *g,* memory, speed, QOL, emotional wellbeing, physical activity, fitness, lack of inflammation, and lack of morbidity, and derived two latent components: Cognitive Wellbeing and Bio-Psychosocial Wellbeing. Although it is not within the aims of PCA to summarize differences among groups, this analysis helped in developing a clearer visualization of the results from LCA. Whereas we had chosen components to represent the three domains a priori, based on their contents, this analysis described the empirical correlational structure of the nine components, which might or might not result in three domains.

## Results

### Formation of wellbeing groups using latent class analysis

Table 1 shows the raw means and standard deviations for the variables used in the first PCA to represent domains of Cognitive Function, Psychosocial Wellbeing, and Physical Health Status. Table 2 shows the BIC and ENT for the LCA models. The BIC suggested a 4- or 5-group solution. ENT indicated that the 2-group solution had the best discrimination amongst the groups. In this study we used LCA as a descriptive tool. We did not expect to find categorically distinct classes; and as such we refer to the results of our LCA as ‘groups’ because it was our judgment that the analyses would not produce exclusively separate categories of individuals but, instead, more loosely-structured groups with indistinct boundaries that would possibly shift amongst samples. Thus, the method had primarily practical and descriptive value, especially since its results could be associated with other variables involved in important life outcomes. We dealt with missing data by using the maximum likelihood estimation missing data feature in MPlus to include all participants.

**Table 1:** Means (standard deviations in parentheses) of the scores on cognitive, psychosocial and physical health domains of wellbeing (n =943 - 1091)

**Components of the domains of wellbeing**		**Range**	**Mean (SD)**
**Cognitive function**	**G**		
	*Symbol search*	2 – 49	24.71 (6.39)
	*Letter-number sequencing*	1 – 21	10.92 (3.16)
	*Matrix reasoning*	4 – 24	13.49 (5.13)
	*Block design*	10 – 65	33.79 (10.32)
	*Digit Symbol Coding*	25 – 98	56.60 (12.93)
	*Digit Span Backwards*	2 – 14	7.73 (2.26)
	***Memory***		
	*Logical Memory I*	0 – 72	43.95 (10.67
	*Logical Memory II*	0 – 48	27.20 (8.23)
	*Verbal Paired Associate I*	0 – 32	19.64 (8.06)
	*Verbal Paired Associates II*	0 – 11	5.95 (2.32)
	***Speed***		
	*SRT (s)*	0.18 – 0.66	0.28 (0.06)
	*CRT (s)*	0.45 – 1.13	0.64 (0.86)
	*IT (total number of correct responses)*	70 – 140	112.1 (11.00)
**Psychosocial Wellbeing**	***Function***		
	*Level of physical activity*	1.1 – 1.5	2.98 (1.1)
	*Days active per month*	0 – 31	7.68 (8.1)
	*ADLs*	0 – 14	1.00 (2.0)
	***Emotional Wellbeing***		
	*HADS (Anxiety)*	0 - 21	4.89 (3.2)
	*HADS (Depression)*	0 – 21	2.80 (2.2)
	*Physical QOL*	0 – 20	16.10 (2.6)
	*Psychological QOL*	0 – 20	15.67 (1.8)
	*Social QOL*	0 – 20	17.14 (2.4)
	*Environmental QOL*	0 – 20	16.71 (1.8)
**Health Status**	***Fitness***		
	*Grip strength (kg)*	5.0 – 55.5	28.8 (10.1)
	*FEV*_*1*_*(L/s)*	0.7 – 4.3	2.4 (0.7)
	*Gait speed (s)*	2.0 – 11.0	1.7 (0.4)
	***Lack of inflammation***		
	*CRP(mg/L)*	1.5 – 45.0	5.26 (6.68)
	*Neutrophil count (10^9/L)*	2.5 – 27.0	4.44 (1.56)
	*Fibrinogen (g/L)*	1.6 – 5.5	3.28 (0.64)
	***Lack of morbidity***		
	*Medical conditions*	0 – 11	2.9 (1.7)
	*Medications*	0 – 8	3.0 (2.5)

*Note. g* = general cognitive ability. ADLs = Activities of Daily Living. HADS = Hospital Anxiety and Depression scales. QOL = Quality of Life. FEV1 = Forced expiratory volume in 1 second. CRP = C-Reactive Protein.

**Table 2:** Model information criteria and entropy for each of the group solutions

**Number of groups**	**BIC**	**ENT**
Two	23501.3	0.826
Three	23334.7	0.746
Four	23258.1	0.771
Five	23200.1	0.769
Six	23174.7	0.759
Seven	23159.2	0.745

*Note.* BIC = Bayesian information criterion. ENT = Entropy.

The 2-group solution consisted of a majority high-wellbeing and an approximately 20% low-wellbeing group; however, this model did not identify some features that were apparent in the 3-group solution. In the 3-group solution, one relatively low-performing group had substantially better cognitive functioning than psychosocial and physical wellbeing, and the other had substantially better psychosocial and physical wellbeing than cognitive functioning. Hence, we identified distinctions in the 3-group solution that were missed in the 2-group solution and which we considered possibly meaningful. Although the 4- and 5-group solutions showed uneven patterns we did not identify further descriptive value than what the 3-group solution produced. In the 3-group solution, the high-scoring group constituted 65.3% (N = 712) of the population, and the two low-scoring groups made up 14.5% (N = 158) and 20.3% (N = 221) of the population. For most likely group membership, the probabilities in the 3-group solution ranged from 0.81 to 0.91, indicating reasonably clear group membership for most participants. The 4-group solution included a subgroup with only 1.4% of the sample, which amounted to only 15 participants, and was unlikely to be robust. This same pattern of subgroups comprising less than 5% of the population also occurred in the 5-, 6-, and 7-group solutions. Since the 2-group solution did not capture the uneven pattern of the 3-group solution, and 4-, 5-, 6- and 7- group solutions included groups representing very small proportions of the sample, we judged that the 3-group model provided the best balance of descriptive value, parsimony, and likely robustness and thus used it for further analysis.

**Figure 2:**
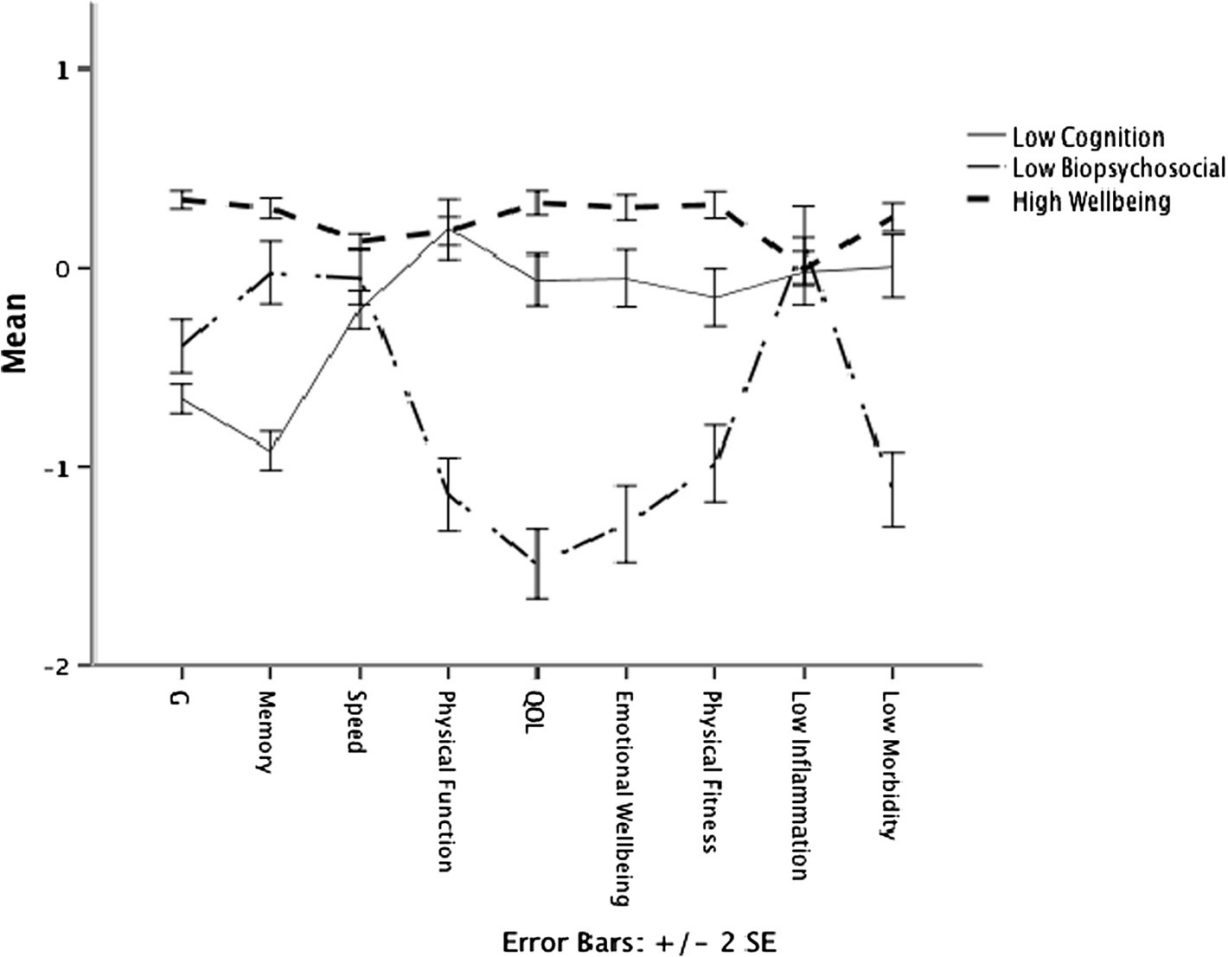
**Mean scores on the nine variables for each of the three groups as generated from latent class analysis with 95% confidence intervals.***Note. g* = General Cognitive Ability. QOL = Quality of Life. For further explanation of the group names’ meanings, see text in the Results section.

We illustrate in Figure 2 how the three groups that emerged from the LCA scored on the full nine components of wellbeing that were assessed. The total number of participants belonging to their assigned group at a probability of .90 or higher can be seen in Table 3.

**Table 3:** Total numbers of participants by group (percentages in parentheses), and numbers of participants with probability of membership at .90 or greater

**Group**	**Total number of cases (%)**	**Number of cases with .90 probability of membership or greater (%)**
1	221 (20.3)	90 (40.7)
2	158 (14.5)	96 (60.8)
3	712 (65.3)	522 (73.3)

**Figure 3:**
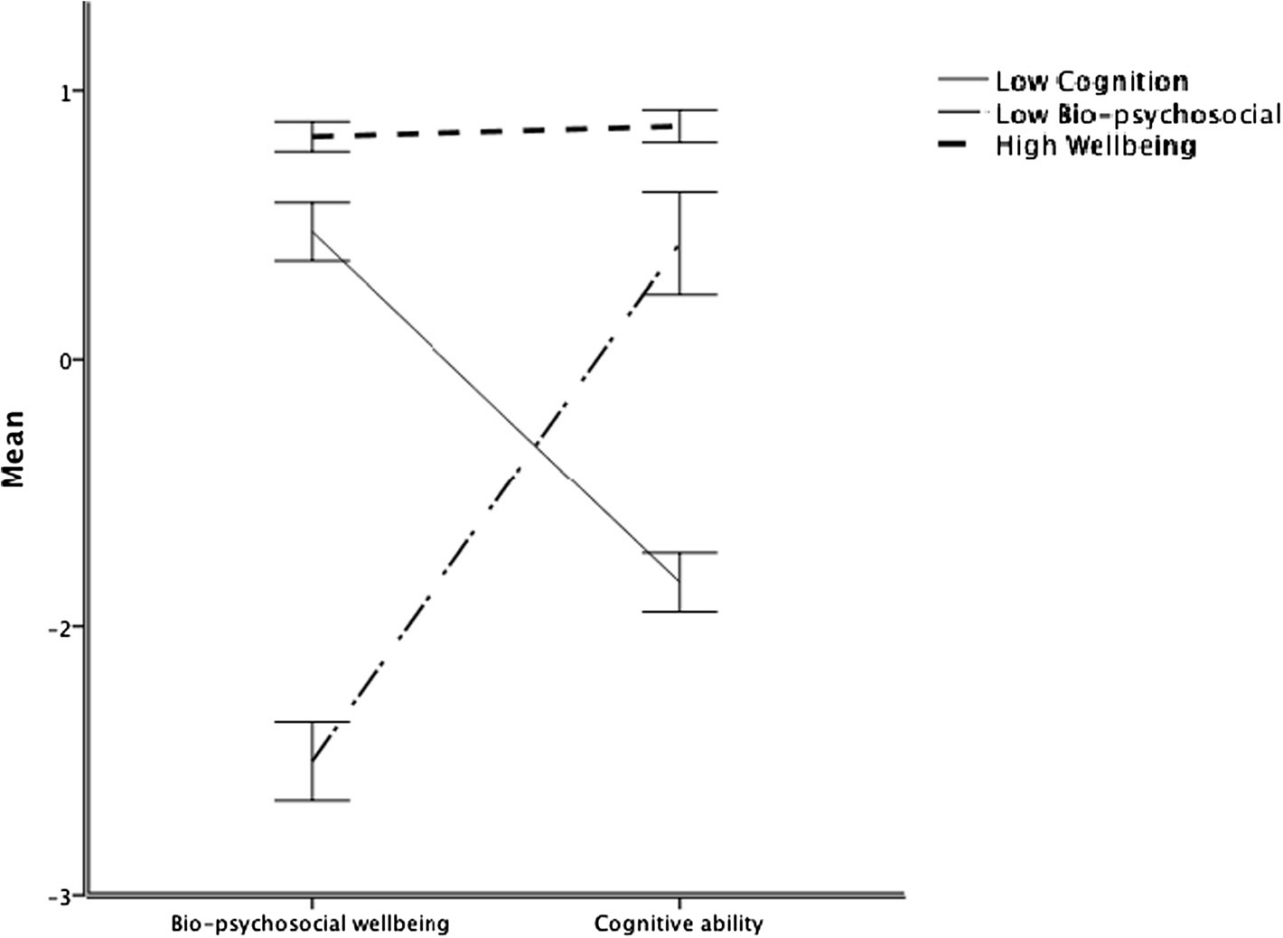
The groups’ mean scores and 95% confidence intervals on Bio-psychosocial Wellbeing, and Cognitive Function as derived from a PCA on the Cognitive Domain, the Psychosocial Domain, and the Physical Domain as derived from LCA for the Lothian Birth Cohort 1936.

To summarize the group features more concisely at the domain level, we submitted the nine variables representing Cognitive Ability, Psychosocial Wellbeing, and Health Status to a single PCA using Varimax rotation. The Kaiser-Meyer Oklin (KMO) measure of sampling adequacy was .68, which is above the acceptable limit of .5 [78]. Examination of the scree plot showed inflexions that justified retaining two components (i.e., not the three domains that we had assumed the nine components would form) explaining 42.1% and 25.0% of the total variance. QOL, Emotional Wellbeing, Fitness, Lack of Morbidity, and Physical Function loaded onto one component, which we labeled the Bio-Psychosocial component. *g,* Memory, Speed and Low Inflammation loaded on another component, which we labeled the Cognitive component. Figure 3 shows the LCA-derived groups’ mean component scores on these two broad domains of wellbeing.

Inspection of Figures 2 and 3, and Table 4, shows that the largest group of individuals scored relatively highly across all variables; we labeled this the High Wellbeing group. One of the other groups (n = 221) had relatively low scores on cognitive ability variables but generally average scores on variables relating to psychosocial wellbeing and health status. The other group (n = 158) had relatively poor scores on psychosocial wellbeing and health status variables, and generally near-average scores on variables reflecting cognitive ability. We labeled these latter two groups Low Cognition and Low Bio-Psychosocial respectively.

**Table 4:** Means (standard deviations and 95% confidence intervals in parentheses) for the derived components of the domains of bio-psychosocial wellbeing and cognitive function for each of the 3 groups of the Lothian Birth Cohort 1936

**Group**	**N (%)**	**Bio-psychosocial wellbeing**	**Cognitive function**
High Wellbeing	712 (65.3)	.33 (0.6, 0.2 – 0.4)	.37 (0.7, (0.3 – 0.4)
Low Bio-psychosocial	158 (14.5)	−2.00 (0.7, −2.2 – -1.9)	-.07 (0.9, 0.3 – 0.1)
Low Cognition	221 (20.3)	-.02 (0.7, −0.1 – 0.1)	−1.34 (0.7, −1.4 – -1.2)

### Associations between wellbeing groups and external variables

Table 5 shows the results from the five groups, including reliability measures of the applicable outcome variables across 2 waves of data (at ages 70 and 73 [79]; of this cohort and internal consistency measures using Cronbach’s alpha [80] for the NEO-FFI for this age-group. The results are described in some detail next.

**Table 5:** External variable means (standard deviations and 95% confidence intervals in parentheses unless otherwise stated as % for categorical variables) and significance values of the differences for each of the three groups

		**Latent groups**					
**Variable**	**T1-T2 (r) or alpha (α)**	**High Wellbeing (n = 712)**	**Low Bio-psychosocial (n = 158)**	**Low Cognition (n = 221)**	***p (HW- LBS)***	***p (HW- LC)***	***p (LBS-LC)***
**Demographics**							
Males	na	346 (48.6)	74 (46.8)	128 (57.9)	< .05	< .05	< .05
Age 11 IQ	na	104.6 (12.3, 103.7 – 105.6)	94.7 (17.2, 91.9 – 97.4)	88.1 (13.8, 86.2 – 90.0)	< .001	< .001	< .001
NART (r)	.96	37.1 (6.8, 36.6 – 37.6)	31.9 (8.2, 30.6 – 33.2)	28.3 (8.0, 27.0 – 29.1)	< .001	< .001	< .001
Years in education	na	11.0 (1.2, 10.1 – 11.1)	10.4 (1.0, 10.3 – 10.6)	10.2 (0.8, 10.1 – 10.3)	< .001	< .001	ns
Still married (%)	na	530 (74.5)	99 (62.7)	149 (67.4)	< .05	< .05	< .05
Living alone (%)	na	157 (22.1)	49 (31.0)	60 (27.1)	< .05	< .05	< .05
Social class I	na	162 (23.0)	15 (9.9)	13 (6)	< .001	< .001	ns
II		282 (40.1)	55 (36.4)	65 (30.2)			
IIIa		163 (23.2)	28 (18.5)	5 (25.6)			
IIIb		77 (10.9)	43 (28.5)	68 (31.6)			
IV		19 (2.7)	7 (4.6)	12 (5.6)			
V		1 (0.1)	3 (2.0)	2 (0.9)			
**Personality**							
Neuroticism (α)	.860	15.4 (6.8, 14.9 – 15.9)	24.5 (7.7, 23.1 – 25.9)	18.0 (7.0, 16.9 – 19.0)	< .001	ns	< .001
Extraversion (α)	.768	27.7 (5.7, 27.3 – 28.1)	23.1 (5.8, 22.0 – 24.1)	27.0 (5.6, 26.2 – 27.9)	< .001	< .001	< .001
Openness (α)	.559	26.8 (5.9, 26.4 – 27.3)	25.0 (5.9, 24.0 – 26.1)	23.9 (4.7, 23.3 – 24.6)	< .01	< .001	ns
Agreeableness (α)	.634	34.0 (5.1, 33.7 – 34.4)	31.6 (5.4, 30.7 – 32.2)	32.6 (5.2, 31.9 – 33.4)	< .001	< .01	ns
Conscientiousness (α)	.797	35.4 (5.7, 35.0 – 35.9)	31.0 (6.7, 29.8 – 32.2)	34.5 (5.5, 33.7 – 35.3)	< .001	ns	< .001
**Health**							
BMI (r)	.95	27.4 (4.1, 27.1 – 27.7)	29.1 (5.1, 28.3 – 29.9)	28.1 (4.4, 27.5 – 28.6)	< .001	ns	ns
Units of alcohol/week	na	11.0 (13.6, 9.9 – 11.9)	9.0 (16.0, 6.5 – 11.5)	10.2 (4.8, 8.3 – 12.2)	ns	ns	ns
Current smokers (%)	na	72 (10.1)	36 (22.8)	38 (17.2)	< .001	ns	ns

Test-retest reliabilities indicated by T1-T2 (r) or validity indicated by Cronbach’s alphas (α) for this age-group are also included where applicable.

*Note.* T1 – T2 = test-retest time 1 – time 2 correlations of the outcome variables indicating reliability across 3 years [79]. NART = National Adult Reading Test. BMI = Body Mass Index. HW = High Wellbeing. LBS = Low Bio-psychosocial. LC = Low Cognition. na = not applicable. ns = not significant.

### Demographics and Prior IQ

ANOVAs showed that The High Wellbeing group had significantly higher age-11 IQ and higher NART scores than the Low Cognition (*d* = 1.26, *d* = 1.19) and the Low Bio-Psychosocial (*d* = 1.19, *d* = 0.69) groups. Individuals in this group also had on average more years in formal education and a higher proportion of individuals belonged to the professional social class (23%) than in the Low Bio-Psychosocial (9.9%) and the Low Cognition groups (6%). The High Wellbeing group had the highest percentage of individuals who were still married (75.0%), and the lowest percentage of divorced individuals (5.8%), compared to the Low Bio-Psychosocial (62.7% and 11.4%, respectively) and the Low Cognition groups (67.4% and 13.1%, respectively). The Low Bio-psychosocial group had the highest percentage of females (53.2%), widowed individuals (17.1%), and individuals who lived alone (31%).There were significantly more males (57.9%) in the Low Cognition group.

### Personality

Results from the ANOVA showed that the High Wellbeing group had significantly higher scores on Extraversion, Agreeableness, and Openness to Experience than the rest of the groups. The High Wellbeing group also had significantly higher scores on Conscientiousness (*d* = 0.7) and significantly lower scores on Neuroticism than the Low Bio-Psychosocial group (*d* = 1.25). The Low Bio-Psychosocial group had significantly lower mean scores on Extraversion (effect sizes up to 0.8), and significantly lower scores on Openness than the other two groups (effect sizes up to 0.54).

### Lifestyle

ANOVA results for the lifestyle variables showed that the High Wellbeing group also had a significantly lower percentage of current smokers (10.1%) than the Low Bio-Psychosocial (22.8%). The Low Bio-Psychosocial group had a significantly higher mean BMI (*d* = 0.37) and a higher percentage of current smokers (22.8%) than the High Wellbeing group, but these did not differ significantly from those of the Low Cognition group.

## Discussion

In this study we investigated whether there were separable patterns of cognitive, psychosocial and physical wellbeing in 70-year-old individuals from the Lothian Birth Cohort 1936. We advanced previous work by simultaneously studying both objective and subjective-based measures of function in three major domains. The results showed a majority of individuals with high wellbeing scores across all domains of function, with two smaller groups showing average scores in some domains but poor scores in at least one. Such majorities of overall good function have been found in several studies including [5,9,11,14,15,24,28,29] typically reflecting the generally good health status of especially older participants who agree to participate in research. The results also showed evidence of the presence of groups with uneven patterns of function. This is possibly because we included psychosocial and emotional wellbeing, which reflect the individual’s adjustment to physical, cognitive and other aging-related changes that are often not within the individual’s control. Self-rated health measures may indicate how individuals believe they are coping with their levels of function as well as the actual levels of function, whilst objective-based measures presumably reflect only individuals’ actual functioning. Two out of three groups displayed uneven patterns of wellbeing, yet the majority of participants were functioning generally well. A group of individuals scored relatively highly on physical health status and psychosocial wellbeing and poorly on the cognitive variables, and another group showed the opposite pattern. These results showed that intelligence and, assuming stability, personality traits seem to influence patterns of health behavior, and ultimately overall wellbeing in old age. Clustering of these variables, which represent three domains of individual differences often considered quite independently, suggested the presence of a unifying underlying dimension that contributed to wellbeing domains in this cohort: the majority scored relatively well across domains of wellbeing and associated co-variables, but those having difficulties appeared to experience them in different areas.

Comparison with our previous research in this sample [14] reinforced that impression. In our prior study, 5 groups were derived based just on self-rated emotional and psychosocial measures, Here, the addition of objectively measured health and cognitive variables characterized the cohort on a wider range of variables commonly depicted in literature on wellbeing in old age. Despite this broader perspective on function we appeared to observe fewer groups yet a similar pattern of function, roughly characterized as most people’s physical and mental functions being quite good comparable, but a few people showing much better function in one area than the other.

One explanation for this particular pattern of uneven results is psychosocial support: when friends and relatives realize that an older person’s cognitive function is slipping, they may be especially likely to offer support sufficient to keep the person physically safe and in reasonably good spirits. In contrast, when cognitive function remains good, both the older individual and friends and relatives may assume the person can and should be taking care of him/herself, despite decreasing energy and overall capability, resulting in loneliness and depression, especially if physical illness sets in.

Others have noted similarly uneven patterns. For example, Morack et al. [24] observed a *compromised memory group* that was characterized by poor cognitive function (specifically memory) but average social and mood scores, although physical fitness was not measured in their study. Our Low Bio-psychosocial group seemed to have similar characteristics to Smith and Baltes’ [29] average profile group 4 labeled *cognitively fit, low extraversion, low control, high social loneliness*. The group in Smith and Baltes [12] study only showed average scores on measures of neuroticism and emotional loneliness (unlike our Low Bio-psychosocial group). Ko et al.’s [11] study also contained one group that had high cognitive function but scored high on Neuroticism measures, showed low perceived social support, though their group was physically fit, while our Low Bio-psychosocial group was not.

Our external set of variables largely reinforced these observations: in a sample of community-dwelling older people such as this one, most people will be functioning pretty well in all areas, but when things start to go wrong, they do so in different ways, dividing especially clearly along cognitive and physical lines. Similarly, in Smith and Baltes’ [12] study, the high performing group seemed to have better levels of performance in other areas of life such as more years in education, better health, and better physical wellbeing, whereas the groups with uneven patterns seemed to showed mixed wellbeing scores such as average levels of wellbeing and low levels of education. This pattern is evident in other cross-sectional studies that studied patterns of wellbeing; e.g. in Ko et al. [11] some profiles showed high marital satisfaction and high scores on measures of depression in the same group, and in Hsu and Jones’ [5] insecure aging group, characterized by good physical and psychosocial function but reported poor economic satisfaction, had high levels of education but reported the lowest life satisfaction out of the four groups found in the study.

Based on the *p* values of group differences, the most important variables that seemed to be distinguishing the groups in this study were childhood IQ, Neuroticism scores, and smoking behavior. Individuals with higher age-11 IQs might have been more able to make better use of their cognitive abilities to engage in health behaviors, make good lifestyle choices, and maintain high levels of wellbeing into old age as evidenced by their high scores on cognitive, physical and psychosocial measures in this study despite the limited educational opportunities at the time this cohort was growing up. Individuals from this sample were born at a time when educational opportunities were limited for many – few had more than three years of education after primary school [81]. Although nowadays educational credentials are often necessary for occupational advancement, this was far less true when many in this sample who had not gone on to further education were at the peaks of their careers, but raw intelligence was often helpful in advancement. There were more opportunities then than there are now to make good incomes in such nonprofessional jobs. Intelligence and well-being associations are typically still observed across many birth cohorts once education and social class are controlled, suggesting significant influence of childhood IQ on later life outcomes [50,51,82]. In fact, higher childhood cognitive ability has been inversely associated with risk of developing cardiovascular disease, vascular dementia, depression, anxiety disorders, posttraumatic stress disorder, substance abuse and comorbid mental disorders [83]. Indeed, lower premorbid IQ scores are related to higher rates of all-cause mortality [49]. The associations between higher childhood IQ and higher scores on wellbeing domains could also arise if cognitive ability is just one more indicator of physiological integrity. This is known as the System-Integrity Hypothesis [52,84,85], which suggests that an efficient brain is a reflection of a “well-put-together” body [50] (p.63), including an efficient brain and robust stress response systems.

As in our prior study, of the five personality traits, scores on Neuroticism distinguished amongst the groups most. The fact that this association was present in both our narrowly focused study and this broader one indicates Neuroticism’s broad reach of influence. Neuroticism is an expression of emotional instability. Like other personality traits, it influences behavior, thus contributing to decision-making and lifestyle patterns, such as smoking behavior [50]. It also influences actions and reactions to daily situations and stressful experiences [86]; thus in a general sense, it is also linked with poor health habits (e.g. high alcohol intake) that may involve self-medication to relieve negative emotions [87,88]. In one study Neuroticism explained between 19% and 88% of covariance among anxiety, depression, and alcohol and substance dependence disorders [89]. Neuroticism has also been associated with health problems such as cardiovascular disease [90], irritable bowel syndrome [91], eating disorders [92], and hypochondriac tendencies [93]. Moreover, it also seems to impact how people cope with disease and treatment regimens, as individuals with high Neuroticism were less likely to adhere to these programs in some studies [94]. In youth Neuroticism is noted to have a protective effect against mortality since individuals high on this trait are more likely to avoid engaging in high-risk behaviour [95]; however, after youth this trait is associated with diminished lifespan [96,97].

The third most notable variable that distinguished the High Wellbeing group from the rest was smoking behavior. This cohort comes from a generation in which smoking was common, even customary, sometimes even especially among the well-off, who could better afford it. It was only in the late 1950s, when individuals from this cohort were in their twenties, that health risks of smoking began to emerge. Thus, smoking behavior in this study reflected to a much larger extent than would be true among young adults today whether individuals who had taken up smoking when they were young followed health advice and quit (43% of the sample), or ignored it and remained current smokers (13% of the sample). The High Wellbeing group had a significantly lower percentage of smokers (10.1%) than the other two groups (17.2% in the Low Cognition and 22.8% in the Low Bio-psychosocial groups). A higher proportion of current non-smokers also belonged to the higher social classes than current smokers, which is reflective of the association we found between social class and wellbeing. In our study an association was present between individuals in the High Wellbeing group and the professional social class. Although the highest proportion of individuals across all groups belonged to the 2nd social class, a higher percentage of individuals in the High Wellbeing group (23.0%) belonged to the professional social class when compared to individuals in the Low Cognition (6%) and Low Bio-psychosocial (9.9%) groups. The implications of these results are that individuals with higher wellbeing are also doing better in society – research shows that childhood IQ predicts adult social economic status [98]. Individuals who are more intelligent may be more cognizant of their career options and further learning opportunities, and be more informed on health and lifestyle decisions, and are in turn better able to exploit their potential [99]. They are also more likely to be living in safer environments and exposed to like-minded individuals in the same social circles who endorse similar values [100].

As with all cohort studies, the historical context of our results needs to be considered as discussed here; however, it is also essential to avoid over-limiting the conclusions to only this cohort, because lifelong intelligence and personality traits are universal traits associated with health behavior and health outcomes [50]. Their significance in overall wellbeing has been documented in previous research [49,101-104] and further strengthened in this study.

### Strengths and limitations

This study had several strengths. The dataset was relatively large at 1091 participants. It contained a wide range of variables, ranging from cognitive ability scores to medical diagnoses, health, physical function, and responses to social questionnaires [51,53]. Variables for group formation were PCA-based composites, reducing error. All participants in the study were born in the same year. This eliminated age-cohort effects within the sample. The overall age-, ethnic, and geographical homogeneity of the participants helped in the identification of groups that were not based on these potentially confounding variables.

The study also had some limitations. The cohort used was relatively healthy, and the study was deliberately aimed at variation in age-related phenotypes among those without severe acute illness. This is common when studying 70-year-old individuals who volunteer for research, and who have been screened for dementia [2,3,29], but the results may be quite far from generalizable to the full population in this age group. The class-solutions selected to describe the groupings of the cohort were based on our judgments of model-fit criteria, entropy, and the importance of parsimony. Although in this study all fit statistics seemed to keep improving with every added group, the group patterns across variables seemed to be getting less parsimonious, with more groups showing similar patterns that could be combined to form more informative groups. This is typical of situations where the data are continuous – although the fit improves with added groups, parsimony decreases, and the chosen group solution is ultimately a matter of the researcher’s informed opinion [105] LCA has been developed to recover qualitative and categorical outcomes [106]; however, it has also been used on continuous data [73], in which the breakdown of natural categories is less likely since the underlying data are dimensional. As in this study, the outcome would be discontinuous since there will have to be some breakpoints to generate groups. However, breaking up a continuum of scores and creating groups out of dimensional patterns of scores can be helpful in summarizing mean patterns of scores from a number of individuals [107]. In this study, where we considered a broader range of function but at less detail, it was revealed with only three groups. This may be because the pattern very clearly featured cognitive function, highlighting its important role in maintenance of wellbeing in old age. Although this study was cross-sectional, the LBC1936 is an ongoing study, with future opportunities to follow up the current results on groups’ stabilities longitudinally to explore the robustness and trajectories of the observed groups. This means that the main effect we focused on could be an artifact, and it might not hold up over time; change measured over just a three-year period is not reliable for meaningful interpretation on patterns of aging and more than two waves of data are required [79]. Still, these results provide descriptive information about the state of function for the participants in the sample at this age. It also serves as a springboard for seeing how results play out for them in the years to come.

## Conclusions

In relatively healthy 70-year-olds, using a number of variables, we found separable groups with respect to objectively-measured and self-reported aspects of wellbeing. The patterns indicated that, for most people, overall wellbeing was quite good, with all aspects as similar levels, but there were distinguishable groups with substantively better wellbeing in some domains than others. Relating the groups to other variables, we found that high trait intelligence, low trait Neuroticism, and not smoking were important predictors of group membership.

Results from this study indicated that although wellbeing in late life seems to be a dimensional process, there are groups of individuals who seem to show uneven patterns of wellbeing, and that possibly such groups need more attention. Awareness of the effect of both intelligence and personality on behavior may improve health interventions and treatment in groups of individuals who show inconsistent profiles of wellbeing. Thus these findings would be useful to carry out in practice: by having researchers educating the public and having them work closely with health-care practitioners and policy-makers to reduce health inequalities. There is increasing interest in the processes of successful aging and there are already emerging fields of cognitive and personological epidemiology that aim to diminish health and wellbeing inequalities by providing care and support as needed [50]. Awareness of individual differences relating to these traits and their behavioral influences on policy-makers and health-care professionals may address clinical issues on risk prevention, improved compliance and better patient-practitioner relationships. The results of this study showed what may contribute to high wellbeing in old age; making this information useful to individuals with poorer outlooks by adopting it in healthcare and policy-making is suggested.

Future work should attempt to replicate these wellbeing groups in other samples and in longitudinal as well as cross-sectional analyses. Mechanisms of wellbeing in each domain—assisted by the evidence about how external variables associated with the domains—can help in understanding how to maximize these desirable states of wellbeing across the life course.

## Competing interests

The authors declare that they have no competing interests.

## Authors’ contributions

ARZ, ID, WJ and JMS have taken part in the designing and planning of the study. ARZ drafted the manuscript, while IJD, WJ and JMS contributed to the pre-submission drafts, revisions and edits of the manuscript. All authors have read and approve the publication of the final manuscript.

## Authors’ note

Andrea R. Zammit, Centre for Cognitive Aging and Cognitive Epidemiology, Edinburgh, EH8 9JZ, UK; Saul B Korey Department of Neurology, Albert Einstein College of Medicine; New York, NY 10461; U.S.A; John M. Starr, Geriatric Medicine Unit, Centre for Cognitive Aging and Cognitive Epidemiology, University of Edinburgh, Edinburgh EH8 9JZ, UK; Wendy Johnson, Centre for Cognitive Aging and Cognitive Epidemiology, Department of Psychology, University of Edinburgh, Edinburgh, EH8 9JZ, UK; Ian J. Deary, Centre for Cognitive Aging and Cognitive Epidemiology, Department of Psychology, University of Edinburgh, Edinburgh, EH8 9JZ, UK.

## Pre-publication history

The pre-publication history for this paper can be accessed here:

http://www.biomedcentral.com/1471-2318/14/53/prepub
